# S-Layer Protein-Based Biosensors

**DOI:** 10.3390/bios8020040

**Published:** 2018-04-11

**Authors:** Bernhard Schuster

**Affiliations:** Institute for Synthetic Bioarchitectures, Department of NanoBiotechnology, University of Natural Resources and Life Sciences, Vienna, Muthgasse 11, 1190 Vienna, Austria; bernhard.schuster@boku.ac.at; Tel.: +43-1-47654-80436

**Keywords:** biosensor, S-layer protein, crystalline 2D protein lattice, lipid membrane platform, linking matrix, bioreceptor, biomimetics

## Abstract

The present paper highlights the application of bacterial surface (S-) layer proteins as versatile components for the fabrication of biosensors. One technologically relevant feature of S-layer proteins is their ability to self-assemble on many surfaces and interfaces to form a crystalline two-dimensional (2D) protein lattice. The S-layer lattice on the surface of a biosensor becomes part of the interface architecture linking the bioreceptor to the transducer interface, which may cause signal amplification. The S-layer lattice as ultrathin, highly porous structure with functional groups in a well-defined special distribution and orientation and an overall anti-fouling characteristics can significantly raise the limit in terms of variety and the ease of bioreceptor immobilization, compactness of bioreceptor molecule arrangement, sensitivity, specificity, and detection limit for many types of biosensors. The present paper discusses and summarizes examples for the successful implementation of S-layer lattices on biosensor surfaces in order to give a comprehensive overview on the application potential of these bioinspired S-layer protein-based biosensors.

## 1. Introduction

**Figure 1:**
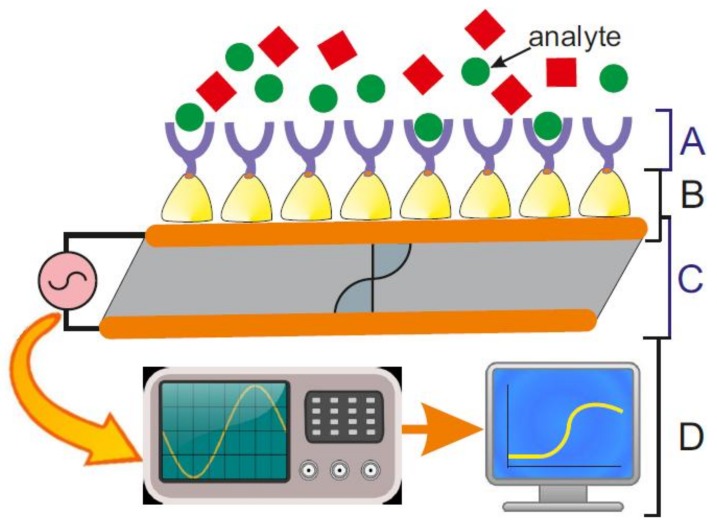
Elements and selected components of an S-layer protein-based quartz crystal microbalance with dissipation monitoring (QCM-D) biosensor. (A) A biosensing element or bioreceptor comprising of accessible functions like, e.g., an antibody to which the analyte binds with highly specific affinity. (B) An interface architecture comprising of a QCM-D sensor surface covered by a recrystallized S-layer lattice, which provides an environment for the proper functioning of the biosensing element. Here, the specific biological event takes place, which gives rise to a certain physical phenomenon. (C) A transducer converting the physical phenomenon (piezoelectricity) resulting from the analyte’s interaction with the biological element into an electrical signals. (D) Associated electronics comprising of signal amplifier, signal processor and a display allowing for a user-friendly visualization and evaluation of the data.

Biosensor-related research has made tremendous progress over the past four decades, because the advance in electronics, nanolithography, nanobiotechnology, biomimetics, and synthetic biology led to successful routes for combining biological systems with silicon technology. Biosensors are per definition devices, which use a biological recognition element that is retained in direct spatial contact with the transduction system [1] or, in simplified terms, a device that converts a physical or biological event into a measurable, mostly electrical signal [2]. In general, biosensors comprise of (Figure 1):

(A) a biosensing element or bioreceptor, to which the analyte has a highly specific binding affinity;
(B) an interface architecture, which provides an environment for the proper functioning of the biosensing element and where the specific biological event, which gives rise to a certain physical phenomenon takes place;
(C) a transducer converting the physical phenomenon or chemical response resulting from the analyte’s interaction with the biological element (e.g., physicochemical, optical, piezoelectric, electrochemical, etc.) into electrical signals. The latter can be reproducibly measured, quantified and processed [3]; and,
(D) an associated electronics comprising of signal amplifier, signal processor and an interface, like a display, which finally allows a user-friendly visualization and evaluation of the data [4].

The biosensing element or bioreceptor is frequently a biologically derived or biomimetic material, like living cell, tissue, enzyme, membrane protein (e.g., ion channel, receptor, pore-forming protein), membrane-active peptide (e.g., ionophore), antibody, nucleic acid, and biological sensitive elements that are created by genetic engineering. The analyte, which binds in a highly specific manner to the bioreceptor may be amongst others ions, nucleic acids, and other organic molecules from cell cultures, human (blood, urine, saliva, tears, sperm, various secretions, etc.) and food samples, and pollutants from environmental samples (e.g., air, water, soil, vegetation).

One of the most challenging tasks is to converge biological or biomimetic systems with silicon technology in order to generate the functional interface architecture [5]. Biological molecules may aggregate or even denature on the surface of electrodes, sensors, or other mostly inorganic solid supports, and hence, loose their function. In order to prevent the loss of function, very frequently an intermediate layer is generated between the biosensing element and the inorganic surface of (ion-sensitive) field-effect transistors, (interdigitated) microarray electrodes, metal-, polymer-, or graphene-coated sensor chips, etc. This intermediate layer comprises either of polymers (e.g., polyethylene glycol, chitosan, agarose, hydrogel, or polyelectrolyte) [6,7], self-assembled monolayers (SAMs; e.g., alkanethiol, dialkylsulfides, silanes, phosphonates) [8,9,10,11], or a monomolecular array of self-assembled protein subunits forming surface layers (S-layers) [12,13,14,15,16]. If a lipid membrane is desired as part of the biosensing element, then an incomplete layer of so-called tether molecules replace the rigid SAM [17,18,19]. Incomplete in the sense that one wants to have only few tether molecules, which anchor the membrane to the surface. This arrangement, where the tether molecules are mixed with so-called spacer molecules ensures a certain retained fluidity of the lipid membrane [18]. The latter is very important if one wants to reconstitute integral membrane proteins and/or membrane-active peptides functionally in a tethered membrane. In general, a tether molecule is composed of a binding group to be anchored on a solid support (thiol, silane, chelation of Ni-ions with nitrilotriacetic acid, biotin, etc.), a hydrophilic backbone, and a hydrophobic moiety to anchor the lipid membrane (alkyl chains, cholesterol, etc.) [20,21]. Molecules, like polymers (in particular polyethylene glycol), glyco-polymers, peptides, and proteins are used so far to build up the hydrophilic part of the tether layers [22,23,24]. The challenge generating the intermediate layer is to combine multiple functions, including: (1) to act as immobilization layer with a suitable binding to both, the inorganic support, and the biological molecules (e.g., bioreceptors, matrix-forming lipids; (2) to allocate a binding matrix where the immobilized molecules are arranged in a well-defined spatial and directed orientation; (3) to provide a reservoir for water and ions; and, (4) to provide sufficient space and stability for the biosensing elements.

Moreover, some biosensors require an immobilization process of the bioreceptor to the sensor surface (metal, metal oxide, glass, polymer, and other materials) using physical or chemical techniques [25]. This is in particular the case if one wants to rely on membrane proteins and membrane-active peptides as biosensing element because these biomolecules need a lipid membrane to adopt their functional structure and to deploy amplification properties. The immobilization of the biosensing element has the additional advantage to be assessable with the broad arsenal of surface-sensing techniques. Indeed, many biosensors rely on surface-sensitive techniques, like surface plasmon spectroscopy (SPR) [26,27,28], surface acoustic wave (SAW), quartz crystal microbalance with dissipation monitoring (QCM-D) [29,30,31,32], electrochemical impedance spectroscopy (EIS) [33,34,35,36], cyclovoltammetry (CV) [37,38,39], or total internal reflection fluorescence microscopy (TIRFM) [40] as transducer. Important questions in this context are how one can create an intermediate layer with all of the intrinsic properties listed above and how can the biosensing element be coupled to or integrated in this functional layer. 

The present paper ocuses on a promising approach to generate a particular type of protein-based intermediate layer, the so-called surface (S-) layer [41]. In the following, I present an introduction to bacterial S-layer proteins and their use for the immobilization of functional molecules and lipid membranes. Moreover, I also introduce S-layer fusion proteins and their utilization as components for the generation of biosensors. Finally, I discuss the application of S-layer lattices for the generation of functional lipid membrane platforms in detail and possible further future directions. 

## 2. Bacterial S-Layer Proteins

**Figure 2:**
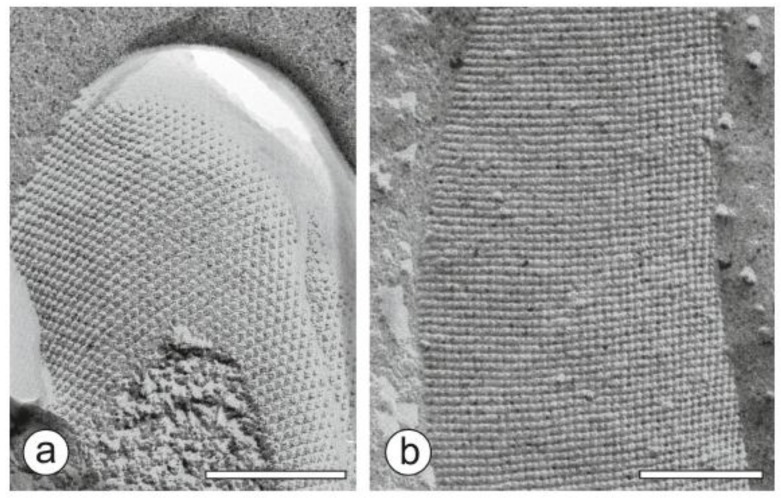
Transmission electron microscopy (TEM) image of a freeze-etched and metal shadowed preparation of (**a**) an archaeal cell (from *Methanocorpusuculum sinense*), and (**b**) a bacterial cell (from *Desulfotomaculum nigrificans*). Bars, 200 nm. Adopted from [15], copyright (2014) with permission from John Wiley & Sons Ltd.

The S-layer is defined as a “two-dimensional array of proteinaceous subunits forming the surface layer on prokaryotic cells” (Figure 2) [15]. S-layers constitute the outermost structure in hundreds of different species of almost every taxonomic group of walled bacteria and are an almost universal feature of archaea [15,42,43,44]. Approximately 10% of the cellular protein mass in bacteria and archaea are S-layers. Hence, the latter are the most abundant biopolymers on Earth [15,45] because the biomass of prokaryotic organisms surpasses the one of eukaryotic organism [46]. Moreover, S-layers also represent the simplest biological protein or glycoprotein membranes that are developed during evolution [14,47,48]. 

High-resolution transmission electron microscopy (TEM) and atomic force microscopy (AFM) studies on the mass distribution of S-layer lattices revealed not only that the S-layer cover the entire cell surface as coherent layer [49,50,51,52], but it also demonstrated the elegancy of these proteinaceous supramolecular bioarchitectures (Figure 2) [53]. Most S-layers are monomolecular assemblies of single subunit species with a molecular weight ranging between 40 kDa to 200 kDa. In general, bacterial S-layer lattices exhibit oblique (p1, p2), square (p4), or hexagonal (p3, p6) space group symmetry with a center-to-center spacing of the morphological units of 3.5 to 35 nm [42,54,55]. Bacterial S-layers are generally 5 nm to 10 nm thick and reveal a rather smooth outer and a more corrugated inner surface. Furthermore, the S-layer lattice SbpA from *Lysinibacillus sphaericus* CCM 2177 showed an outstanding antifouling characteristic of in the presence of highly concentrated protein solutions (e.g., 70 g L^−1^ human serum albumin), plasma, and whole blood samples [56]. This finding is explained by the inherently (zwitterionic) neutral charge of the outer surface of SbpA. Moreover, S-layers are highly porous protein lattices (30% to 70% porosity) with pores that are uniform in size and morphology in the dimension of 2 nm to 8 nm [57,58,59]. Interestingly, many S-layers possess two or even more distinct classes of pores [42,54,55,60,61].

Little is known about the specific biological functions of S-layers, but it is now evident that they can function as protective coats against, e.g., bdellovibrios, bacteriophages, and phagocytosis; can act as molecular sieve, molecule, and ion traps; promoters for cell adhesion; immune-modulators; surface recognition; antifouling coatings; and, virulence factors in pathogenic organisms [15,58,62,63]. Moreover, the S-layer lattice is important for the determination of cell shape and as a structure that is aiding in the cell division process in archaea [64,65]. Interestingly, microbial S-layer protein arrays from *Deinococcus radiodurans* show ion-gating properties [66]. Ion transport appears to be mainly due to an electrical gradient inside the pores, which most probably originate from negatively charged amino acid side chains. The evaluation of the gating characteristics toward various ion species suggests that these S-layer protein arrays constitute a biological ion gate with calcium selectivity [66].

One very important feature of S-layer proteins is the capability of isolated native or recombinantly produced subunits to self-assemble into crystalline arrays on surfaces or interfaces. These surfaces include mica, silicon nitride, silicon oxide, glass, noble metals, like platinum, gold, titanium, stainless steel, but also many polymers, like cellulose, polyester, polystyrene, and technically relevant materials, including graphene, indium tin oxide, and highly oriented pyrolytic graphite [56,67]. TEM [68,69,70] and AFM [71,72,73] are the most appropriate techniques to elucidate the recrystallization process of S-layer proteins. Crystal growth at interfaces (e.g., solid supports, air-water interface, or lipid membranes) starts simultaneously at many randomly distributed nucleation points. Subsequently, it proceeds in plane until the crystalline domains meet, thus leading to a closed, coherent mosaic of individual, several micrometer large S-layer patches [71,74,75,76]. A low monomer concentration, which corresponds to a low number of nucleation sites, favors the growth of extended S-layer patches. The monocrystalline, individual patches are separated by grain boundaries [71]. 

The formation of a coherent crystalline lattice depends on the used S-layer protein species, the environmental conditions of the subphase (i.e., ionic content and strength, pH-value), and on the surface properties of the interface. Interestingly, S-layer lattice can exhibit against cells in tissue cultures either cell adhesive (cytophilic) or cell repulsive (cytophobic) surface properties, depending whether the inner or outer side, respectively, faces the aqueous environment. Adjusting the recrystallization protocol from a basic condition (pH 9; resulting in an exposed outer, smooth cytophobic side) to an acidic condition (pH 4; resulting in an exposed inner rough, cytophilic surface pattern) results in the different orientation, and thus, function of the S-layer protein lattice [77].

While the reassembly of S-layer proteins at the air-water interface and at planar lipid films is well defined [69,70,78,79,80], the deliberate modification of the surface properties of a solid support allows for specifically controlling the reassembly process [71,75,81,82,83]. For example, the S-layer protein SbpA, which is one of the most extensively studied S-layer proteins for functionalizing solid supports, forms monolayers with a height of 9 nm on the hydrophobic surface. In contrast, SbpA forms on hydrophilic silicon supports a double layer with an interdigitated-toothed rack-like structure with a height of 15 nm [71]. Furthermore, in comparison to hydrophilic surfaces, the layer formation is much faster on hydrophobic supports because it starts from many different nucleation sites, and thus, leads to a mosaic of small crystalline domains [47]. 

In general, the S-layer is, on the one hand side, utilized as very precise immobilization matrix to present various biomolecules, including bioreceptors, in a unique manner [15,84,85]. On the other hand side, this protein-based intermediate layer constitutes also a versatile base plate for the generation of supported lipid membranes, which provide the essential environment for the reconstitution of functional membrane proteins and membrane-active peptides [41,86,87].

Great potential as patterning elements and nanoscale building blocks is evident for native S-layer proteins. However, genetic approaches open up the possibility of modifying and tuning the natural properties of S-layer proteins [88,89]. Nevertheless, one has to take care that the S-layer proteins comprising inserted or fused foreign functions or protein domains retrain their capability to self-assemble into geometrically well-defined layers. The most relevant advantages of genetically engineered S-layers over less nanostructured approaches are the periodicity of functional domains in the nanometer range. To date, the generation of S-layer fusion proteins occurs through the homologous expression and the secretion by cells or inside a host organism, which is, in most cases, *Escherichia coli* [89,90]. Both of the strategies result in bio-inspired materials with designed functional properties. Moreover, the possibility to fuse single or multifunctional domains of other proteins to S-layer proteins opens up a broad spectrum of applications ranging from biosorption of heavy metals, formation of nanoparticle arrays, vaccine development, over immobilized biocatalysts, fluorescent biomarkers, and diagnostic tools to sensor development [15,50,91,92]. In this context, it is interesting to note that S-layer fusion proteins presenting domains for the covalent binding of lipid molecules constitute a very promising strategy to enhance the stability of the so-called S-layer supported lipid membrane (SsLM) [93,94,95,96,97]. Finally, co-recrystallization of different S-layer fusion proteins will lead to a high flexibility for the variation of functional groups within a single S-layer array.

## 3. Modified S-Layers as Components in Biosensors

S-layers represent one of the few examples in nature, where proteins reveal the intrinsic capability to self-assemble into monomolecular lattices. In general, the S-layers represent an ideal patterning element for nanobiotechnological and biomimetic applications [41,50,52,98,99,100]. Particularly, the repetitive physicochemical properties and isoporosity of this proteinaceous lattice structure down to the sub-nanometer scale make them to unique matrices and building blocks. The prime attractiveness of such ‘bottom-up’ strategies is not only their capability to assemble into uniform nanostructures, but also the possibility to exploit these structures at the meso- and macroscopic scale. Moreover, the cloning and characterization of genes encoding S-layer proteins opens new areas for applied S-layer research as the incorporation of single or multifunctional domains is now feasible without the loss of their self-assembly capability [88]. However, as S-layers significantly differ in their inner and outer surface with regard to their topography and physicochemical properties, it is essential to ensure a correctly oriented recrystallization of S-layer (fusion) proteins on solid supports and lipid membranes [71,77]. Hence, the biomimetic approach copying prokaryotic cell envelope structures with their intrinsic properties might be among others a distinguished solution for the formation of supporting scaffolds for lipid membrane platforms.

The recognition of biosensors started with the introduction of the first generation glucose oxidase (GOx) biosensor in 1962 [101], which is still the most widely used biosensor up to date [102,103,104,105]. Electrochemical biosensors, as exemplified by the glucose sensor, do not suffer from the drawback of high sensor setup complexity and costs because of their close link to the developments in low-cost production of microelectronic circuits and their simple interface with standard electronic read-out and processing units. Moreover, electrochemical biosensors are robust, easy to miniaturize, can be used in turbid biofluids containing optically absorbing and fluorescing compounds, and show excellent detection limits, even when operating with small analyte volumes [103,106,107]. 

For the generation of a GOx biosensor it proved very beneficial to relay on the isoporous S-layer-lattice structure as molecular pin board [108,109,110,111]. For this purpose, S-layer ultrafiltration membranes (SUMs) are produced by depositing S-layer-carrying cell wall fragments or S-layer self-assembly products on microfiltration membranes, crosslinking the S-layer protein under a certain pressure, and finally reducing the Schiff bases [109,112]. Beside enzymes (GOx, invertase, peroxidase, glucuronidase, β-glucosidase, naringinase), also ligands (protein A, folate, streptavidin), or mono- and polyclonal antibodies have been immobilized on SUMs [12].

Since S-layers constitute an immobilization matrix of only several nanometers thickness, the fabrication of unsurpassed thin sensing layers with densely packed functional biomolecules, in particular enzymes, is possible [113,114,115,116]. The first amperometric sensor based on an S-layer lattice comprised of an SUM with covalently bound GOx [117]. The retained activity of the immobilized GOx was approximately 40%. In order to function as working electrodes, a layer of gold or platinum covered the enzyme loaded SUMs. The biosensor yields high signals (150 nA mm^−2^ mmol^−1^ glucose), fast response times (10–30 s), linearity range up to 12 mM glucose, stability under working conditions of more than 48 h, and no loss of GOx activity after a storage period of six months. A further achievement with S-layer-based amperometric biosensors was the generation of a three-enzyme sensor for sucrose [118]. For this purpose, the enzymes invertase, mutarotase, and GOx were immobilized on S-layer fragments that were isolated from *Clostridium thermohydrosulfuricum* L111-69 via aspartic acid as spacer molecules. After the deposition of the modified S-layer fragments on microfiltration membranes, the surface of this multifunctional device was covered with gold by sputtering to function again as a working electrode. Amperometric sucrose measurements that were based on the oxidation of hydrogen peroxide revealed a high signal level (1 µA cm^−2^ mmol^−1^ sucrose), 5 min response time, and a linear range up to 30 mM sucrose. A further development resulted in a glucose sensor with an oxygen optode as transducer containing a ruthenium (II) complex, whose fluorescence is dynamically quenched by molecular oxygen [119]. For the fabrication of this fibre-optic biosensor, the GOx was covalently immobilized as a monolayer on SUMs. The performance of this biosensor in terms of response time, linear range, and stability was comparable to existing optodes. However, this system holds great potential for the development of micro-integrated optical biosensor due to its tiny size. A further improvement of this fibre-optic glucose sensor was to connect the GOx molecules in its tightest packing immobilized on the S-layer lattice with an optimum metallic contact, which must not disturb the protein structure [120]. Hence, platinum films, being applied by means of argon sputtering covered the enzyme layers immobilized on the S-layer protein. However, this conventional method exhibits substantial limitations, e.g., a volume change of the S-layer/enzyme composite system when it is introduced into a conventional vacuum coating apparatus. The application of the pulse-laser-deposition method circumvented this drawback. The latter approach resulted in an enzyme activity of 70–80%, which constitutes a doubling of the activity compared to first amperometric sensor that was based on an S-layer lattice [114,117,120]. Hence, this example demonstrates that the composite system consisting of the metal/enzyme/two-dimensional (2D)-protein layer arrangement can successfully serve as highly efficient biosensors.

The accurate on-line detection of glucose in blood is a challenging task because many blood components disturb the measurement. As S-layer lattices constitute ultrathin and highly hydrated materials [121], Picher et al. designed a lab-on-a-chip comprising of embedded amperometric sensors in four S-layer-coated micro-reactors, which can be addressed individually [56]. The S-layer had the function to provide an efficient antifouling coating, a highly-oriented immobilization matrix for the GOx, and an effective molecular sieve. Moreover, the S-layer protein SbpA readily formed monomolecular lattice structures on the surface of various microchips (e.g., gold, glass, polydimethylsiloxane, platinum) within 60 min. The microfluidic device operated in a feedback loop mechanism in order to assess the natural variations in blood glucose levels during hemodialysis, and thus, to allow for the individual adjustment of glucose. To ensure reliable and accurate detection of glucose in blood the lab-on-a-chip performed simultaneously blood glucose measurements, mediator-interferences detection, background subtractions, and auto-calibration routines. The highly isoporous SbpA-coating eliminated unspecific adsorption events in the presence of freshly drawn blood samples, human serum albumin, and plasma. Most important, the undisturbed diffusion of the mediator to the electrode surface enabled electrochemical measurements of glucose in the range from 0.5 mM to 50 mM [56]. Hence, this combination of biologically derived nanostructured surfaces with microchip technology constitutes a powerful tool for the multiplexed analysis of complex samples.

The bioreceptor in cholesterol biosensors is very commonly the cholesterol oxidase (ChOx). A simple and reliable method to prepare reproducible and stable ChOx monolayers was to spread the ChOx at the water–air interface. Mixed films comprising of ChOx and S-layer proteins showed a long-term stability at the air-water interphase [122]. In a further study, the mixed film was transferred onto the surface of screen-printed carbon electrodes by the Langmuir-Blodgett technique [123]. Characterization of the modified electrode surface occurred with AFM and cyclic voltammetry (CV). AFM indicated the presence of deposited layers, which also resulted in a reduction of the surface roughness of the electrodes. As demonstrated by CV, the presence of S-layer proteins in the ChOx Langmuir-Blodgett film increased the oxidation peak intensity and reduced the oxidation potential. Therefore, these results showed the feasibility of producing a cholesterol biosensor that was based on the immobilization of a mixed film comprising of ChOx and S-layer proteins on screen-printed carbon electrodes [123]. 

For the development of oxygen sensors, an oxygen sensitive Pt(II) porphyrin dye was covalently bound to the S-layer matrix [124]. Setups comprising of low cost optoelectronic components, like light emitting diodes and silicon photodiodes, measured the oxygen concentration by phase modulation fluorimetry. Variations in the oxygen concentrations resulted in distinct and reproducible changes in the luminescence lifetime and intensity for planar and fiber optic sensor setups. The luminescence quenching efficiency of these sensors was found to be 1.5–1.9 (expressed as the ratio of signal under nitrogen and air), which compares well to other sensor systems using luminophores that are embedded in polymer matrices. These results demonstrated the application potential of S-layers as immobilization matrices in the development of biosensors [124]. Hence, the general principle for the construction of optical sensors by immobilization of various dyes, fluorophores and/or receptors on ultrathin S-layer protein coatings led already to methods for the sensing of manifold analytes.

In a recent study, a monomolecular S-layer lattice comprising of the S-layer protein SbpA that was conjugated with folate was recrystallized on a gold surface [39]. This biorecognition layer ensured the specific capture of human breast adenocarcinoma cells (MCF-7) via the recognition of folate receptors, which are expressed on the surface of MCF-7. The fabricated acoustic and electrochemical sensors were able to distinguish between MCF-7 and human liver hepatocellular carcinoma (HepG2) cells as the latter do not express folate receptors. This biosensor offers several advantages, including the small thickness of the SbpA lattice, which increases cells’ capturing efficiency. Moreover, there is no requirement to block the surface due to the antifouling properties of the S-layer lattice and no awareness of antibody immobilization as folate can be used as an alternative to the antibody for capturing target cells. Several techniques provided evidence for the efficiency of this biosensor. QCM-D measurements tracked the formation of SbpA-folate modified sensor and the capturing of cancer cells efficiently in real-time and under controlled conditions. Although the QCM-D technique shows a limited detection range, it allows for tracking the cell viability [39]. Hence, the cellular response to chemotherapeutic agents is worth to be investigated in further QCM-D studies. Indeed, electrochemical measurements confirm the selectivity and the specificity of the developed biosensor and provide a simple, rapid, cost-effective, and disposable analysis of cancer detection. Moreover, the development of efficient biosensors for accurate diagnosis helps to increase the cure and the survival rates of patients with cancer and provides great promise for an effective analysis with high selectivity and sensitivity.

In another study, chemical crosslinkers linked a thrombin-binding aptamer and an ofloxacin-binding aptamer to different to S-layer proteins isolated from *L. sphaericus* JG-A12 and *L. sphaericus* JG-B53. [125]. S-layer protein monomers were not able to crystallize after aptamer modification and showed no thrombin binding during random surface attachment. In contrast, aptamers that were linked to an intact S-layer in suspension or an S-layer coating were still functional. Laser-induced fluorescence spectroscopy, resonant mirror sensory, and QCM-D verified the functionality of both aptamers through target binding after S-layer immobilization on solid supports [125].

The S-layer protein from *L. sphaericus* JG-A12 was bound on a gold surface in order to fabricate a uranyl (UO_2_^2+^) biosensor. Immobilization occurred either by the binding of the cysteine of the S-layer protein to a SAM, which presented maleimide groups or to a mixed SAM presenting biotin, which bound neutravidin and the latter subsequently to the biotinylated S-layer protein [126]. The biosensor responded to picomolar levels of aqueous uranyl ions within minutes and showed higher stability and longer electrode life span in comparison to traditional SAM-based biosensors. The biosensors detected specifically UO_2_^2+^-ions with a detection limit of 10^−12^ M. Chemical modification of the phosphate and carboxyl groups of the S-layer protein prevented UO_2_^2+^ binding, indicating that both of the moieties are involved in the recognition to UO_2_^2+^ [126]. The same S-layer protein, was adsorbed on gold nanoparticles. The functionalized gold nanoparticles aggregated in the presence of arsenic species and resulted in a color change from burgundy-red for widely dispersed nanoparticles to blue for aggregated nanoparticles. By this means, a concentration of the anionic arsenic (V) complex lower than 24 ppb was detectable [127]. In future, it might be possible that S-layer protein isolates from bacteria surviving in other metal polluted sites may provide the sensing components for the fabrication of further biosensors for the detection of other metal ions.

## 4. Genetically Engineered S-Layers as Components in Biosensors

Their intrinsic self-assembly properties as well as their periodicity make S-layers ideal building blocks for all kinds of detection systems, like DNA-, aptamer-, protein-, allergy-, or antibody-chips, as well as label-free detection systems (for review see [15,52]). 

The construction of S-layer-streptavidin fusion proteins carrying core-streptavidin either at the N-terminus or C-terminus allowed for the generation of universal affinity matrices for the specific binding of biotinylated molecules like, e.g., proteins, allergens, antibodies, oligonucleotides, or nanoparticles [89,128]. Another application potential is in the development of label-free detection systems. The specific binding of functional molecules to the sensor chip functionalized with an oriented chimaeric S-layer can be measured directly by determining the change in mass on the chip. In addition, there is no need for any labeling if the applied transducer relies upon surface-sensitive techniques, like QCM-D, SPR, or SAW. 

Proof-of-principle for label-free detection systems based on S-layer proteins was performed with the S-layer fusion protein incorporating the sequence of a variable domain of a heavy chain camel antibody directed against prostate-specific antigen (PSA) [129,130]. A monomolecular lattice comprising of S-layer fusion protein recrystallized on gold chips constituted the sensing layer in SPR biochips to detect PSA. A further application for this chimaeric S-layer fusion protein was to recrystallize them on silica microbeads. These protein-covered microbeads constituted the biocompatible matrix at a microsphere-based detoxification system that was used for extracorporeal blood purification of patients suffering from autoimmune disease [131].

In another approach, the recombinant S-layer fusion protein rSbpA/ZZ that was incorporating two copies of the Fc-binding Z-domain, which is a synthetic analogue of the IgG-binding domain of protein A from *Staphylococcus aureus* was constructed. Most importantly, the ZZ-domains remained exposed on the outermost surface of the S-layer fusion protein lattice. As determined by SPR measurements, the binding capacity of the self-assembled rSbpA/ZZ monolayer for human IgG was 5.1 ng/mm^2^, which corresponded to 78% of the theoretical saturation capacity of a planar surface for IgGs aligned in the upright position [132]. Cellulose-based microbeads with recrystallized rSbpA/ZZ S-layer fusion protein on the surface constitute a novel detoxification system. The IgG binding capacity of the S-layer fusion protein-coated microbeads was at least 20 times higher when compared to commercial particles that were used as immunoadsorbents to remove autoantibodies from sera of patients suffering from an autoimmune disease [132]. Very recent developments are an acoustic biosensor and a hybrid three-dimensional printed electrochemical biosensor both based on rSbpA/ZZ for the detection of liver cancer cells [37]. The biosensors function by recognizing the highly expressed tumor marker CD133, which is located on the surface of liver cancer cells. The S-layer protein rSbpA/ZZ presenting the ZZ-domain enabled the immobilization of highly accessible anti-CD133 antibodies as a sensing layer for the efficient detection of HepG2 cells. QCM-D and CV measurements provided evidence for the recognition of HepG2 cells in situ and confirmed the efficiency of the fabricated sensors to perform label-free and real-time detection of living cells. Most importantly, these sensors offer disposable and low-cost detection platforms for real-world applications, e.g., for large-scale clinical and drug-screening studies [37].

For another field of research, S-layer fusion proteins comprising of SbpA or SbsB, the S-layer protein from *Geobacillus stearothermophilus* PV72/p2 and peptide mimotopes, such as F1, which mimics an immunodominant epitope of Epstein-Barr virus (EBV) were constructed [133,134]. The screening of 83 individual EBV IgM-positive, EBV-negative, and potential cross-reactive sera resulted in 98.2% specificity and 89.3% sensitivity, as well as no cross-reactivity with related viral diseases. The result of this diagnostic study disclose the performance of these S-layer fusion proteins as a matrix for site-directed immobilization of small ligands in solid phase immunoassays.

Finally, the laccase of *Bacillus halodurans* C-125 was immobilized on the S-layer lattice that was formed by SbpA either by covalent linkage of the enzyme or by construction of a fusion protein comprising the S-layer protein and the laccase (rSbpA/Lac) [135]. TMeasurements on the specific activity of the free, immobilized, and fused showed for all laccase-like activity by oxidizing 2,2′-azino-bis(3-ethylbenzthiazoline-6-sulfonic acid), 2,6-dimethoxyphenol, syringaldazine, and hydroquinone. Interestingly, the S-layer part imparts a much higher solubility of the laccase when compared to the sole enzyme. Spectrophotometric measurements of the enzymatic activity revealed similar but significantly higher values for laccase and rSbpA/Lac in solution as compared to the immobilized state, respectively. However, laccase covalently linked to the SbpA monolayer yielded a four- to fivefold higher enzymatic activity than rSbpA/Lac immobilized on a solid support. Combined QCM-D and electrochemical measurements revealed that the laccase that was immobilized on the SbpA lattice had an approximately twofold higher enzymatic activity when compared to that obtained with rSbpA/Lac [135].

## 5. S-Layer Lattices for Generation of Functional Lipid Membrane Platforms

**Figure 3:**
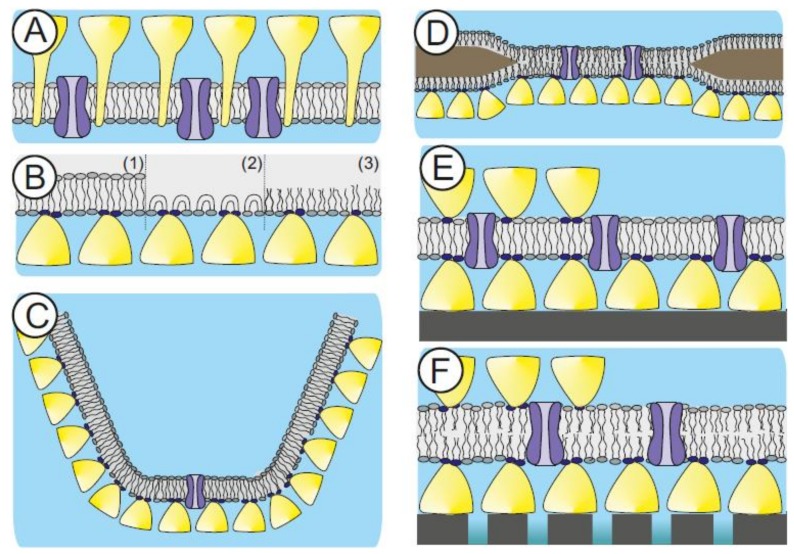
Scheme of natural and surface layer (S-layer) supported lipid membranes. Supramolecular structure of an archaeal cell envelope comprising of a cytoplasma membrane, archaeal S-layer proteins incorporated in the lipidic matrix and integral membrane proteins (**A**). Schematic illustrations of various S-layer-supported lipid membranes. (**B**) Lipid monolayer films at the air/water interphase with a recrystallized S-layer lattice underneath. (1) Tetraether lipid monolayer in the upright conformation. (2) Tetraether lipid monolayer in the U-shaped (bent) conformation. (3) Phospholipid monolayer. (**C**) A tetraether lipid monolayer membrane is generated across an orifice of a patch clamp pipette by the tip–dip method. Subsequently a closely attached S-layer lattice is formed by bacterial S-layer proteins on one side of the lipid membrane. In (**D**), a folded or painted bilayer phospholipid membrane spanning a Teflon aperture is shown. A closed bacterial S-layer lattice can be self-assembled on either one or both (not shown) sides of the lipid membrane. (**E**) Schematic drawing of a solid support where a closed bacterial S-layer lattice has been assembled. On this biomimetic structure, a tetraether lipid membrane was generated by the modified Langmuir-Blodgett method. Optionally as shown on the left side, a bacterial S-layer lattice can be attached on the external side of the solid supported lipid membrane. (**F**) Scheme of a bilayer lipid membrane generated on an S-layer ultrafiltration membrane. Optionally as shown on the left side, a bacterial S-layer lattice can be attached on the external side of the S-layer ultrafiltration membrane (SUM)-supported lipid membrane. In B to F, the head groups of the lipid molecules interacting with the S-layer protein are marked in dark. As indicated in C to F, all S-layer-supported model lipid membranes can be functionalized by biomolecules like membrane-active peptides and integral membrane proteins. Modified after [63], copyright (2004) with permission from Wiley-VCH.

The cell envelope structure of some archaeal species (e.g., *Sulfolobus* spp.) are made of a cytoplasma membrane comprising of etherlipids and a membrane-anchored S-layer lattice (Figure 3A) [44,136]. In a biomimetic approach, this supramolecular cell envelope structure constitutes the building plan for SsLMs (Figure 3B–F). It is assumed that the cell envelope structure of archaea is a key prerequisite for these organisms to be able to dwell under extreme environmental conditions, such as temperatures up to 120 °C, pH down to 0, high hydrostatic pressure, or high salt concentrations [42,137,138]. Hence, S-layer lattices may therefore be very important to provide basic functions, like mechanical and osmotic cell stabilization [139,140]. So far, S-layer proteins from Gram-positive bacteria are used in the generation of SsLMs because suitable methods for the disintegration of archaeal S-layer protein lattices and their reassembly into monomolecular arrays are not available [41,93,95,97,98,141]. In addition, a second S-layer as a protective molecular sieve, stabilizing scaffold, and antifouling layer can be self-assembled on the top of the SsLM (Figure 3E,F). These features make S-layer lattices unique supporting architectures, resulting in lipid membranes with nanopatterned fluidity and considerably extended longevity [41,93,94,95,97,98,142,143,144].

The interaction of the S-layer proteins SbpA and SbsB with lipid molecules has been investigated in detail [78,79,80,145]. It turned out that most probably negatively charged moieties on the S-layer protein interact via electrostatic interaction with the head groups of zwitterionic and/or positively charged lipids (Table 1). As natural, wild-type S-layer proteins frequently possess a so-called S-layer homologous domain, which interacts with the secondary cell wall polymer, the latter can be coupled to the head group of a lipid and the lipid can be immobilized via a lectin-type like binding on the S-layer protein [18]. The recrystallized S-layer protein can also be chemically modified in order to bind lipids with head groups comprising of a primary amine group [146,147], thiol group [148], or maleimide group (Table 1). Moreover, a linker, like, e.g., streptavidin, can be coupled to the S-layer protein, which allows for a strong ligation of biotinylated lipids [149,150]. Finally, genetically engineered S-layer proteins may expose either a thiol group from an introduced cysteine, multiple histidine residues (His-tag), streptavidin, or a strept-tag (Table 1). These modifications allow for the coupling of lipids carrying a maleimide group [151,152,153], nickel(II)-nitrilotriacetic acid anchor [154], or biotin at their head group region [73,155,156], respectively.

**Figure 4:**
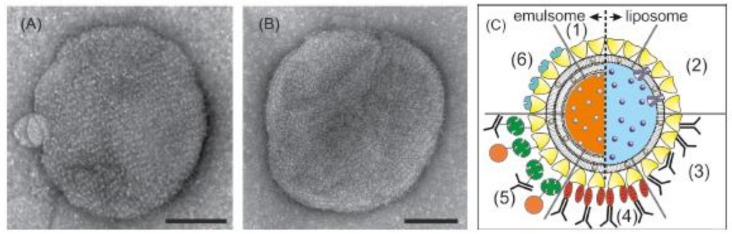
S-layer-coated liposomes and emulsomes. TEM images of emulsomes coated with the S-layer protein SbsB from *Geobacillus stearothermophilus* PV72/p2 (**A**) wildtype SbsB and (**B**) recombinant SbsB. The bars correspond to 100 nm. Adopted from [191], copyright (2013) with permission from Wiley-VCH. (**C**) Schematic drawing of (1) an S-layer coated emulsomes (left) and Iiposome (right) with entrapped functional molecules and (2) functionalized by reconstituted integral proteins. Note, S-layer coated emulsomes can only transport hydrophobic molecules but with a much higher transport capacity. S-layer coated emulsomes and liposomes can be used as immobilization matrix for functional molecules (e.g., human IgG) either by direct binding (3) or by immobilization via the Fc-specific ligand protein A (4), or biotinylated ligands can be bound to the S-layer coated liposome or emulsomes via the biotin—avidin system (5). Alternatively, emulsomes or liposomes can be coated with genetically modified S-layer subunits incorporating functional domains (6). Modified after [61], copyright (2002) with permission from Wiley-VCH.

In general, S-layer proteins can be recrystallized on either a lipid monolayer generated at the air-water interface (Figure 3B), a preformed lipid membrane like a planar, freestanding lipid bilayer (Figure 3C,D), or a spherical liposome or emulsome (Figure 4). Moreover, the lipid membrane can be generated on an already existing recrystallized S-layer lattice (Figure 3E,F). The latter approach is the method of choice to generate functional lipid membrane platforms, which are, beside other applications, a straightforward approach in the development of biosensors [41,144].

Fluorescence recovery after photobleaching measurements determined the fluidity of SsLMs prepared by the Langmuir-Blodgett/Langmuir-Schaefer technique, silane-supported, and dextran-supported phospholipid bilayer [157]. The highest nanopatterned fluidity of the lipids showed the SsLMs as compared to the other supported bilayers. This is most probably due to the repetitive local interaction of the S-layer lattice with the lipid head groups. In another study, it was feasible to generate phospholipid bilayers and tetraether lipid monolayers on S-layer covered gold electrodes. A very exciting result was that the tetraether lipid monolayer with an S-layer lattice on both sides (Figure 3E) revealed an exceptional long-term robustness of approximately one week [41,93,96,97,98].

The generation of lipid membranes on porous supports combines the advantage of easy manual handling, individual excess to both membrane surfaces, and possessing an essentially unlimited ionic reservoir on each side of the bilayer lipid membrane (BLM; Figure 3F). However, the ultrastructure of the porous support, like roughness or strong heterogeneity in the size of the pore diameters, has a significant impact on the stability of the attached BLM [158]. Hence, a straightforward approach is the utilization of SUMs with the S-layer acting as a stabilizing and anchoring structure and as a smoothening layer, which reduces the roughness of the porous support [159,160,161].

S-layer fragments deposited as a coherent layer on microfiltration membranes are forming the SUM [109,111,162]. The mechanical and chemical stability of the SUM is subsequently introduced by chemical inter- and intramolecular cross-linking [109,110,162,163,164]. The S-layer lattice with its uniformity of functional groups on the surface and within the pore area permits the very accurate chemical modifications in the sub-nanometer range. These modifications enable the tuning of the molecular sieving as well as antifouling characteristics of SUMs [162,163,165]. Moreover, SUMs can be prepared with different net charges and hydrophobic or hydrophilic surface properties. These features make SUMs very attractive as stabilizing and supporting structures for the functional lipid membranes [15,41,94,97,144].

SUM-supported phospholipid bilayers were highly isolating structures with a lifetime of up to 17 h [159,160,161], whereas BLMs on plain microfiltration membranes revealed a life-time of only approximately 3 h. The lifetime increased significantly to about 24 h by the attachment of an S-layer lattice on both sides of the lipid membrane (Figure 3F) [159,160]. The crosslinking of those lipid head groups, which are direct in contact with domains on the S-layer protein may result in a further increase of the longevity of this composite membrane architecture, and is thus a promising strategy for generating stable and fluid lipid membranes [86].

Investigations on the incorporation behavior of the membrane-active peptides valinomycin, alamethicin, gramicidin D, and the negatively charged antimicrobial peptide analogue peptidyl-glycine-leucine-carboxyamide PGLa(-) permit drawing conclusions on the functionality of SsLMs resting on solid supports (Table 2) [159,166,167]. SsLMs with incorporated valinomycin, which is a potassium-selective ion carrier, exhibited in sodium buffer a remarkable high membrane resistance. However, the change to potassium buffer resulted in a decrease of the membrane resistance by a factor of 500 because now the valinomycin-mediated potassium transport could take place [159].

Combined surface-sensitive QCM-D and EIS measurements elucidated the attachment and/or insertion of PGLa(-) in SsLMs that was generated by the rapid solvent exchange technique. The results of this study indicated toroidal pore formation in a concentration-dependent manner [168]. In addition, electrochemical measurements on gramicidin, which is a membrane-active peptide (Table 2), revealed the formation of functional gramicidin pores in all of the mentioned SsLMs [161]. Moreover, the SsLMs allowed for tracing of even single gramicidin pores. Thus, SsLMs are promising lipid membrane platforms for studying the interaction and insertion of membrane-active (antimicrobial) peptides in model lipid membranes [169,170].

Finally, SsLMs provided also a proper matrix for the functional incorporation of alamethicin (Table 2). The addition of amiloride, which is an inhibitor for alamethicin, resulted in a specific blocking of the alamethicin channels as increasing amounts of amiloride gave rise to a significant increase in membrane resistance [159]. Thus, this result provided proof-of-concept for the applicability of these composite S-layer/lipid structures for biosensing purposes. In future, the ability to reconstitute membrane-active peptides like antimicrobial ones in defined structures on, e.g., sensor surfaces is of high importance for the design and fabrication of biomimetic sensing devices [41,171,172,173].

The reconstitution of integral membrane proteins in SsLMs was also successful (Table 3). For example, α-hemolysin (αHL), which is a pore-forming heptameric protein formed functional pores in SUM-supported phospholipid bilayers but no pore formation was achieved for BLMs that were generated on plain microfiltration membranes. Interestingly, with SUM-supported lipid membranes, even single αHL pore recordings are feasible [160].

The reconstitution of the integral ryanodine receptor, RyR1 (Table 3), which was isolated from rabbit muscle cells was also successful in SsLMs [174]. For formation of SsLMs by the newly developed β-diketone ligand europium-triggered vesicle fusion technique [147], resulted in isolating lipid membranes. Depending on the type of measurement, the SsLM formation occurred either on glass surfaces (for fluorescence experiments) or on gold sensors (for QCM-D measurements). Preliminary measurements clearly indicated that incorporation of RyR1 took place. This was verified by control experiments to exclude misinterpretation due to unspecific adsorption of RyR1 to the bilayer or the S-layer lattice [174]. Nevertheless, further experiments, like, e.g., combined QCM-D with EIS studies on SsLMs or patch clamp measurements on a chip, will help to implement SsLMs as a versatile and stabilizing scaffold for detailed studies on the impact of different drugs on reconstituted RyR1 in high-throughput screening-like devices.

The addition of the voltage-dependent anion channel (VDAC; Table 3) to a preformed SsLM caused a significant decrease in membrane resistance, whereas the membrane capacitance did not change significantly [147]. Moreover, an increasing VDAC concentration led to a decrease of the membrane resistance, indicating an increasing number of spontaneously reconstituted VDAC channels in the SsLM [175]. VDAC is a voltage-gated channel, which is at high membrane potentials in the closed state and switches to an open state at low membrane potentials (less than10 mV) [176,177,178]. Indeed, VDAC channels that are reconstituted in SsLMs clearly showed the above described behaviour. Furthermore, the membrane resistance decreased again after reducing the voltage from 10 mV back to zero, but the resistance remained higher when compared to the first measurement. The reason for this may be the re-opening of some channels while others remain closed. In addition, it is conceivable that keeping the channels in the closed state for a long period during the measurements may reduce the rate of re-opening of VDAC and cause some structural rearrangements in order to achieve a more stable closed conformation [177,179]. Moreover, the presence of the nucleotides nicotinamide adenine dinucleotide hydride (NADH) or nicotinamide adenine dinucleotide phosphate hydrogen induce channel closure, leading to a significantly reduced conductance of the VDAC channels [180,181,182,183]. Indeed, the addition of NADH to the VDAC channel reconstituted in SsLM caused a significant increase in the membrane resistance, which strongly evidenced the blocking of VDAC channels by NADH molecules [175].

All the before mentioned examples for the functional reconstitution of membrane-active peptides and membrane proteins in SsLMs are currently proof-of-principle studies. There is, however, a strong desire to use SsLMs, particularly for probing the function of membrane proteins, e.g., in drug screening applications. The fact that membrane proteins currently comprise more than 50% of all drug targets and many of these membrane proteins are directly involved in various transfer processes across the membrane demonstrate the importance of performing detailed studies on the structure and function of membrane proteins [184,185]. A direct electrical readout of membrane functions, e.g., of ionic currents generated by ion channels, provides the advantage of a signal amplification. There is no need for any labelling, as it is mandatory for many current state-of-the-art techniques that are used for membrane protein screening.

Vesicular lipid structures, like unilamellar liposomes, comprising of a closed, spherical lipid mono- or bilayer with an aqueous inner space and emulsomes, comprising of a solid fat core that is surrounded by lipid layers are mainly used as drug targeting and drug delivery systems [186,187]. However, these lipid nanoparticles can also be used as biosensors for diagnosis purposes if the drug is replaced or is supplemented by a radiotracer, contrast agent, or a fluorescent dye. The use of molecular imaging to non-invasively measure the in vivo distribution of nanomedicines becomes increasingly important [188]. Labeling the nanoparticle gives also an indication of delivery to the target tissue. In this context, it is worth mentioning that liposomes and emulsomes can be covered by an S-layer lattice (Figure 4A,B) [150,189,190,191,192]. In addition, the S-layer lattice may be functionalized with, e.g., antibodies in order to detect cancer cells (Figure 4C). Moreover, S-layer-coated and with labelling agents loaded liposomes or emulsomes may be used for molecular imaging to detect, e.g., inflammations in a body.

## 6. Conclusions & Outlook

A highly challenging scientific area is the research at the intersection of biological and engineering sciences for the development of biosensors. Significant progress in issues like miniaturization, functional sensitivity, simplified read-out, multiplexing, and the utilization of newly discovered physical phenomena further pushed the development of smart devices. Moreover, semiconducting technology has proceeded in a way that in the field of biosensors a rapid infiltration of new (bio)nanotechnology-based approaches occurred. Due to bottom-up self-assembly processes at the nanometer scale, the traditional separation between transducers and bioreceptors is not valid anymore. Indeed, by an integrative approach, the interface architecture plays an important role in the recognition event and the receptors become active transducing elements of the biosensors (Figure 1).

The present paper describes the successful implementation of cell envelope components, like, e.g., phospho- and etherlipids, and in particular, bacterial S-layer proteins in biosensors. S-layer proteins can be self-assembled to become part of the interface architecture and thereby connecting the bioreceptor to the transducer interface. The recrystallized S-layer lattice provides significant advantages over other coatings, which can be summarized as follows: (1) S-layers are very thin structures (5 nm to 10 nm). This becomes very important if one uses surface-sensitive phenomena as transducer, as it is indicated in Figure 1. Because these techniques have a limited measuring range, the sensitivity decreases with an increasing intermediate layer thickness. (2) S-layers are highly porous structures (30% to 70% porosity). This becomes very important if one uses electrochemical methods as transducer. Due to the electrolyte-filled pores, there is much less limitation of ions and the S-layer lattice itself shows a negligible resistance and capacitance. (3) The S-layer lattice presents functional groups in a well-defined special distribution and orientation. This property allows for arranging bioreceptors as densely packed and highly directed on the interface. (4) The S-layer lattice shows an anti-fouling, self-cleaning surface where almost no biomolecules stick to it. This characteristic is favourable when the measured signal corresponds to the bound and adsorbed biomolecules on the sensor surface. Finally, (5) the S-layer lattice can completely cover areas in the cm^2^-range by a one-step process. Hence, a coherent proteinaceous coating can functionalize the whole surface of commonly used transducers. Although only little material is necessary, a possible drawback of using S-layer proteins is the need for the scale-up of the cultivation of bacteria and the isolation, subsequent purification, and the storage of S-layer proteins. However, recombinant production of S-layer proteins may help to overcome this issue. Moreover, physicists have to consider adopting certain protocols to meet the demands of biological materials.

As previously mentioned, because of their structural features, S-layer lattices are highly suitable to immobilize biosensor-relevant molecules, like enzymes, dyes, fluorophores, and receptors. In another approach, the bioreceptor may be fused to the S-layer protein. The S-layer protein recrystallizing part ensures a layer presenting the bioreceptor molecules, like enzymes, antibodies, IgG-binding domains, and peptide mimotopes in a tight packing and rectified orientation. The S-layer lattice is used also as an anchoring scaffolding and/or ion reservoir in the generation of lipid membrane platforms. In contrast to tethered lipid membranes, where a precisely balanced mixture of tether and spacer molecules have to be assembled on the sensor surface, only one type of biomolecules, the S-layer protein, is sufficient to provide few repetitive anchoring points for the lipid membrane.

Moreover, biologically inspired lipid membrane-based platforms enabled the unprecedented signal amplification down to single-molecule sensitivity. This was achieved by the creation of mechanically and chemically stable membrane platforms with a high longevity. A further crucial property of membrane platforms is their ability to host membrane-associated and -integrated biomolecules like membrane-active peptides, ionophores, pore-forming proteins, ion channels or (G-protein coupled) receptors in a functional form. All these, in many cases highly sensitive biomolecules distinguish themselves by operating at very low concentrations of, e.g., ligands. For instance, biological nanomachines, like G-protein coupled receptors and ion channels, are very successful in solving the problem of selective and efficient amplification of binding events.

Miniaturization of membrane platforms in a chip format reduces the volume of needed biological material and allows for very sensitive recording of single membrane protein activities. Miniaturized membrane platforms are very promising in the field of drug discovery due to the possibility to directly record membrane protein functionality when they are exposed, e.g., to lead compounds and drug candidates. At present, the research on biosensor is not only driving the ever-accelerating race to construct devices that are more efficient, smaller, and cheaper, but may also ultimately result in the successful integration of electronic and biological systems, and hence, in novel electronic sensing technologies.

## Figures and Tables

**Table 1:** Immobilization strategies. Summary of the to date investigated strategies to bind lipid molecules on S-layer protein lattices (modified after [41]).

Type	Reactive Group	Crosslinker	Targeted Group	References
**Natural SPLs**				
Electrostatic interaction	Negative charges on SLP		Zwitterionic or positively charged lipids	[79,80,145]
Lectin-type like binding	S-layer homologous domain on SLP		Secondary cell wall polymer coupled to lipids	[18]
**Chemical modi-fication of SLPs**				
Covalent bond	Carboxyl groups on SLP	Carbodiimide analogues	Primary amine group from lipids	[146,147,168]
Covalent bond	Primary amino groups on SLP	SMCC analogues	Thiol group from lipids	[148]
Covalent bond	Primary amino groups on SLP	SPDP/TCEP; insertion of thiol group in SLP	Maleimide group from lipids	Schuster et al. in preparation
**Chemical binding of linker on SLPs**				
Strong ligation	Streptavidin chemically coupled to SLP		Biotinylated lipids	[149,150]
**Genetically engineered SLPs**				
Covalent bond	Thiol-group from introduced cysteine		Maleimide group from lipids	[151,152,153]
Multiple chelation	Multiple histidines (His6-tag) on SLP		Nickel(II)-NTA from lipids	[154]
Strong ligation	Streptavidin fused to SLP		Biotinylated lipids	[89]
Strong ligation	Strep-tag fused to SLP	Streptavidin	Biotinylated lipids	[72,73,155]

SLP: S-layer protein; SMCC: Succinimidyl-4-(*N*-maleimidomethyl)cyclohexane-1-carboxylate; SPDP: *N*-Succinimidyl 3-(2-pyridyldithio)-propionate; TCEP: Tris (2-carboxyethyl) phosphine hydrochloride; NTA: nitrilotriacetic acid.

**Table 2:** Membrane-active peptides. Summary of membrane-active peptides incorporated in S-layer supported lipid membranes (modified after [41]).

Membrane-Active Peptide	Source	Remarks	References
gramicidin A(gA)	*Bacillus brevis*	linear pentadeca peptide	[161]
alamethicin(Ala)	*Trichoderma viride*	linear, 20 amino acids	[159]
valinomycin (Val)	several *Streptomyces* strains, e.g., *S. tsusimaensis* and *S. fulvissimus*	cyclic dodecadepsi peptide, 12 amino acids and esters	[141,159]
peptidyl-glycine-leucine-carboxyamide (PGLa) analogue	synthesized via protein chemistry	20 amino acid; negatively charged analogue of PGLa	[168]

**Table 3:** Transmembrane proteins. Summary of transmembrane proteins reconstituted in S-layer supported lipid membranes (modified after [41]).

Transmembrane Protein	Source	Remarks	References
α-hemolysin (αHL)	exotoxin from *Staphylococcus aureus*	pore-forming; homo-heptamer	[144,160]
ryanodine receptor 1 (RyR1)	skeletal muscle cells	Ca^2+^-release channel; homotetramer	[174]
nicotinic acetylcholine receptor (nAChR)	plasma membranes of neurons; on postsynaptic side of the neuromuscular junction	ligand gated ion channel; 5 subunits	[154]
voltage-dependent anion channel (VDAC)	located on the outer mitochondrial membrane; also produced by cell-free expression	porin, voltage gated; ion channel monomeric but can cluster	[175]

## Abbreviations

2D	two-dimensional
αHL	α-hemolysin
AFM	atomic force microscopy
BLM	bilayer lipid membrane
CD133	tumor marker
ChOx	cholesterol oxidase
CV	cyclovoltammetry
EBV	Epstein-Barr virus
EIS	electrochemical impedance spectroscopy
GOx	glucose oxidase
HepG2	human liver carcinoma cells
IgG	immunoglobulin G
MCF-7	human breast adenocarcinoma cell
nAChR	nicotinic acetylcholine receptor
NADH	nicotinamide adenine dinucleotide hydride
PGLa(-)	negatively charged analogue of peptidyl-glycylleucine-carboxyamide
PSA	prostate-specific antigen
QCM-D	quartz crystal microbalance with dissipation monitoring
RyR1	ryanodine receptor/Ca^2+^ release channel
rSbpA/Lac	recombinant fusion protein comprising of SbpA and laccase
rSbpA/ZZ	recombinant fusion protein comprising of SbpA and two copies of the Fc-binding Z-domain (a synthetic analogue of the IgG-binding domain of protein A from *Staphylococcus aureus*)
SAM	self-assembled monolayer
SAW	surface acoustic waves
SbpA	S-layer protein of *Lysinibacillus sphaericus* CCM 2177
SbsB	S-layer protein of *Geobacillus* *stearothermophilus* PV72/p2
S-layer	two dimensional arrays of proteinaceous subunits forming surface layers on prokaryotic cells
SPR	surface plasmon resonance
SsLM	S-layer supported lipid membrane
SUM	S-layer ultrafiltration membrane
TEM	transmission electron microscopy
TIRFM	total internal reflection fluorescence microscopy
VDAC	voltage-dependent anion channel
